# National initiatives to promote quality of care and patient safety: achievements to date and challenges ahead

**DOI:** 10.1186/s13584-020-00417-x

**Published:** 2020-11-05

**Authors:** Dalia Dreiher, Olga Blagorazumnaya, Ran Balicer, Jacob Dreiher

**Affiliations:** 1 Free International University of Moldova, Chisinau, Moldova; 2 Clalit Health Services, Tel Aviv, Israel; 3 Faculty of Health Sciences, Ben-Gurion University of the Negev, Beer Sheva, Israel; 4 Soroka University Medical Center, Beer Sheva, Israel

**Keywords:** Quality improvement, Patient safety, Quality indicators, Accreditation, National programs

## Abstract

**Background:**

The quality of healthcare in Israel is considered “high”, and this achievement is due to the structure and organization of the healthcare system. The goal of the present review is to describe the major achievements and challenges of quality improvement in the Israeli healthcare system.

**Body:**

In recent years, the Ministry of Health has made major strides in increasing the public’s access to comparative data on quality, finances and patient satisfaction. Several mechanisms at multiple levels help promote quality improvement and patient safety. These include legislation, financial incentives, and national programs for quality indicators, patient experience, patient safety, prevention and control of infection and accreditation. Over the years, improvements in quality indicators, infection prevention and patient satisfaction can be demonstrated, but other fields show little change, if at all. Challenges and barriers include reluctance by unions, inconsistent and unreliable flow of information, the fear of overpressure by management and the loss of autonomy by physicians, and doubts regarding “gaming” of data. Accreditation has its own challenges, such as the need to adjust it to local characteristics of the healthcare system, its high cost, and the limited evidence of its impact on quality. Lack of interest by leaders, lack of resources, burnout and compassion fatigue, are listed as challenges for improving patient experience.

**Conclusion:**

Substantial efforts are being made in Israel to improve quality of care, based on the use of good data to understand what is working and what needs particular attention. Government and health care providers have the tools to continue to improve. However, several mechanisms for improving the quality of care, such as minimizing healthcare disparities, training for quality, and widespread implementation of the “choosing wisely” initiative, should be implemented more intensively and effectively.

## Background

The Israeli healthcare system is characterized by universal health coverage through the 1995 National Health Insurance Law (NHIL). Primary and secondary care is provided by four health plans, based on the Bismarckian model [1]. The four health plans, or HMOs, are non-profit and funded by the government. Hospital services are provided by the government (50% of acute hospital beds), health plans (mostly Clalit Health Services, the largest of them, which operates a third of acute hospital beds), and other private and public owners [2]. All health plans and hospitals have sophisticated information systems that include electronic medical records and data on activity levels, services provided and quality of care; there are also several systems for aggregating data across providers, including national registries for conditions such as cancer and diabetes and reporting of cases of infectious diseases. The government uses regulation to promote access to care, quality of care, financial stability and equity [3].

The goal of the present review is to describe the major achievements and challenges of quality improvement in the Israeli healthcare system. We focus on the main mechanisms utilized to improve quality. These include legislation, national programs for quality indicators, financial incentives accreditation and training. We also focus on specific topics such as reducing health disparities, infection prevention and control, patient safety and patient-centered care. We conclude by describing the major barriers to quality improvement and some concluding remarks. While our main goal is to describe current mechanisms available for improving quality, some historic perspective (from 2000 to the present) is also provided.

## Methods

For this review, we surveyed PubMed for studies published within the last 10 years with any of the keywords “quality indicators”, “patient experience”, “patient safety”, “accreditation” and “health disparities” plus the word “Israel”. Studies in either English or Hebrew were included. Overall, 1037 studies were identified, of which we chose 29 studies for this review. We also used Google to search for non-peer reviewed publications by the Ministry of Health (MOH), Myers-JDC-Brookdale, the Israeli National Institute for Health Policy Research, and the Oreganization for Economic Cooperation and Development (OECD) (Mainly circulars, official reports, etc.)

Wherever possible, we extracted data from various publication to compare performance over time in quality indicators, health disparities, patient safety, infection prevention and patient satisfaction over time, Since data are provided for different years in different topic, some of which have data from the 1970s, while others were measured only recently, we needed a consistent method to compare performances over time. We defined a significant improvement as a 10% improvement over 5 years, or an annual mean improvement rate of 2.1%, in closing the gap to 100%. Therefore, for indicators with a potential 100% goal (“high is good”) the mean annual rate of improvement from a performance level of P_1_ in the first year (Y_1_) to a performance level of P_2_ in the most recent year Y_2_, was defined as

$$ {\left(\frac{\mathbf{1}\mathbf{00}\%-{\boldsymbol{P}}_{\mathbf{2}}}{\mathbf{1}\mathbf{00}\%-{\boldsymbol{P}}_{\mathbf{1}}}\right)}^{\frac{\mathbf{1}}{\left({\boldsymbol{y}}_{\mathbf{2}}-{\boldsymbol{y}}_{\mathbf{1}}\right)}} $$

For indicators with a potential 0% goal (“high is bad”) the mean annual rate of improvement from a performance level of P_1_ in the first year (Y_1_) to a performance level of P_2_ in the most recent year Y_2_, was defined as

$$ {\left(\frac{{\boldsymbol{P}}_{\mathbf{2}}}{{\boldsymbol{P}}_{\mathbf{1}}}\right)}^{\frac{\mathbf{1}}{{\boldsymbol{y}}_{\mathbf{2}}-{\boldsymbol{y}}_{\mathbf{1}}}} $$

### Overview of quality in Israel

The quality of healthcare in Israel is considered “high”. This achievement can be attributed to the universal health coverage guaranteed by the NHIL of 1995, and to the structure and organization of the healthcare system, which utilizes a robust primary care system, high professional standards of clinicians and supportive factors for quality such as medical education and research. However, some challenges face the Israeli healthcare system. In a paper published in 2001 [4], several such challenges were listed, including accessibility of services and long waiting lines, inefficiency in some services, lack of community and continuity between primary care and hospital care, overload of medical teams, avoidable harm, and barriers to effective communication between caregivers and patients [4]. To this day, these are the key challenges in the Israeli healthcare system. To these challenges we can add widening of health disparities and increasing out-of-pocket cost, as well as burnout of medical teams: physicians and nurses show a burnout score of 3.6 and 3.5, respectively, on a 1–5 scale, where score more than 3 are considered high [5], compared to a score of 2.2 in a survey of 20,000 employees in Israel conducted in the last decade.

In recent years, the Ministry of Health (MOH) has made transparency one of its main goals and it has made major strides in increasing the public’s access to comparative data on quality, finances and patient satisfaction. These advances in transparency have also facilitated greater accountability, both to the government and to the general public. National surveys show that three quarters of patients are satisfied with their hospital care and that almost all Israelis are satisfied or very satisfied with their health plans overall (~ 90%). Life expectancy is higher and mortality is lower than the Organization for Economic Cooperation and Development’s (OECD) average, but Israel ranks in the mid-range among European countries for the statistic called, “mortality amenable to health care” [6–8], Israel’s standing relative to the OECD average is mixed with regard to avoidable hospitalizations and in-hospital mortality rates, while rates of hospital-acquired infections are higher than in many other developed countries. The quality of the primary care provided by the health plans has been found to be good both in comparison with United States plans and from a broader international perspective [2].

### Legislations and bylaws

The OECD claims that the MOH has “an eclectic range of tools at its disposal”. The MOH grants licenses to most health care facilities, inspects them and investigates complaints. Through enforceable “directives”, the MOH can compel public and private hospitals to comply with certain procedures and it maintains regular dialogue with the four health plans on addressing gaps and improving quality [9].

The two main laws regarding quality in Israel are the National Health Insurance Law (NHIL) of 1995, and the Patient’s Rights Law of 1996. The NHIL vaguely references the quality of care, stating that “healthcare services included in the ‘basket’ of medical services will be offered based on medical discretion, with reasonable quality, in a reasonable timeframe and at a reasonable distance from the place of residence of the insured person” (article 3d). The law refrains from dealing with the appropriateness of resources, review of processes and outcomes, way of monitoring and reporting, etc. [4, 10]. An extensive review of this point is brought in the report of the recent Dead Sea Conference (2019) which focused on “25 years to the National Health Insurance Law: challenges and modifications needed for the coming decade” [11].

Porath [4] suggested enacting a National Quality in Health Care Law, which would define the National Quality Improvement Institute, a government-sponsored body. Its roles would include evaluating the quality of care in several fields, with public reporting as a major role along with oversight of quality improvement initiatives [4]. While such an institute was never established, the Quality and Services Wing of the MOH takes on some of these roles, including the management of the National Program for Quality Indicators for Hospitals, performing national quality surveys, leading the National Program for Patient Safety, improving patient experience in the Israeli healthcare system, managing complaints and public queries, and improving internal services to the MOH.

Over the years, several regulations and MOH directives have been published. The National Health Insurance Regulations (Quality Indicators and Data Disclosure) of 2012 specify the basis for the National Quality Indicator Program for Hospitals. These regulations state that the MOH’s Director General, after consulting with an advisory committee, will form a list of quality indicators which will be published on the MOH’s website. The advisory committee includes representatives from the MOH, health plans, academy, and hospitals not owned by health plans. Public reporting is to be made transparent, i.e. identifying the name of provider to which the data refers [12]. Medicine Division directive 22/2014, “The National Program for Quality Indicators in Hospitals: Monitoring and Follow-up” [13] defines “excellence” and “failure to meet goals” related to the national targets, and the various sanctions for hospitals which consistently fail to meet the national goals. Other directives form the basis for hospital accreditation by the Joint Commission International (Medicine Division directive 38/2012 [14], see below); define the structure of the Unit for Patient Safety in hospitals and health plans (Medicine Division directives 35/2012 [15] and 48/2013 [16], respectively); and define a list of “never events” (Director General directive 09/2011 [17]) and adverse events which need to be reported to the MOH (Medicine Division directive 11/2012 [18]).

### National programs for quality indicators

For 15 years, Israel has had an extensive and successful program for monitoring quality of care in the community. This program was first developed in Clalit Health Services, the largest of the four health plans, as of 1998 [19]. The National Program for Quality Indicators in the Community started in 2003 as a joint research venture of the Faculty of Health Sciences and the Faculty of Management in Ben-Gurion University of the Negev. In 2004, this program was announced by the MOH as a national program, and since then it operates based on voluntary cooperation of the four health plans. An annual report is published following review by an external auditor [10, 19].

In recent years, that program has undergone several important developments, first including some comparative performance data by health plans in 2011, stratified by age groups and socioeconomic status to such a degree that direct comparison between health plans became almost impossible, and finally incorporating explicit comparisons into the report as late as 2016.

Following the project’s success in advancing the quality of care in primary care, more than 10 years ago, the MOH established it nationally under the supervision of the National Institute for Health Policy Research. This program now enjoys the full support and voluntary cooperation of all four health plans and is led by a separate academic directorate. The National Program for Quality Indicators in the Community Healthcare maintains a measure of the quality of primary care and assesses health, wellness, and disease management through 76 quality indicators in nine major clinical domains: health promotion, cancer screening, child and adolescent health, health in adults aged 65 years or older, respiratory diseases, cardiovascular health, diabetes, antibiotic use and mental health. Data are systematically collected for the entire population, based on the electronic health records of the four health plans. The program uses benchmarking to national programs for quality indicators in other countries, including the United States, Sweden, Australia, the United Kingdom and the OECD. Criteria used for selecting quality indicators are importance and relevance, evidence-base, quantification, feasibility and the ability to implement changes. Types of measures include epidemiological measures, process measures and outcome measures. In 2018, the report covered 8.3 million enrolees [3, 20]. A review by the OECD described this program as “one of the most sophisticated programs to monitor the quality of care in primary care across OECD countries” [9]. Many of the program’s indicators are based on definitions from existing international measures, such as those in the Healthcare Effectiveness Data and Information Set (HEDIS) of the National Committee for Quality Assurance (NCQA) in the United States, and with the intention of international comparison [9]. Jaffe et al. [21] reviewed the improvement in quality indicators over time. Similarly, studies on the improvement in diabetes care [22, 23] and the elderly [24] have been published. Improvements in diabetes care in the community were associated with improved health. Specifically, these achievements consisted of accelerated decreases in lower limb amputations in men and in mortality due to diabetes in the Arab population [22]. In a study focused on the elderly population [24], gender disparities were observed across all measures, with worse indicator rates among females compared to males. Socioeconomic disparities were not consistent across measures. Of note, Jewish-Arab differences were not investigated in this study.

When looking at performance over time (Table 1) major improvements in performance rates can be demonstrated in many indicators. However, when looking carefully at the data, it seems that most improvements happened up to 2014 (90% of indicators have improved up to this point), and than the performance level reached a plateau and has not improved since 2014 (only 13 indicators, 36%, show substantial improvement since 2014).

**Table 1:** Performance in quality indicators in the community over time [20, 25–27]

Indicator	First Year	Performance in the First Year	Performance, 2014	Performance, 2018
Influenza vaccine, asthma	2003	23.1%	**41.7%**	35.3%
Asthma medication ratio > 0.5	2014		67.4%	**70.9%**
Spirometry in COPD	2013	52.0%	**57.9%**	**75.5%**
Mammography	2003	51.6%	**69.3%**	**72.5%**
Colorectal screening	2003	11.8%	**58.3%**	**64.8%**
PAP smear, 3y	2014		48.1%	51.5%
Influenza vaccine >65y	2003	43.9%	**63.4%**	60.4%
Pneumococcal vaccine	2008	70.9%	**76.6%**	77.5%
Periodic testing for Hb A1c in diabetes	2003	85.2%	**90.0%**	90.7%
Hb A1c - good control	2003	42.0%	**66.4%**	**70.8%**
Ab A1c > 9%	2003	17.8%	**11.6%**	**9.6%**
LDL in diabetes	2003	83.5%	**90.8%**	90.9%
LDL < 100 in diabetes	2003	39.2%	**63.1%**	66.0%
Eye exam in diabetes	2003	56.5%	**75.0%**	72.5%
Microalbumin tested	2003	38.8%	**78.6%**	**81.2%**
Influenza vaccine, diabetes	2003	38.6%	**61.6%**	59.8%
BP documented in diabetes	2003	38.7%	**90.0%**	90.7%
BMI documented in diabetes	2008	81.8%	**88.0%**	84.8%
Pneumococcal vaccine, diabetes	2008	76.3%	**81.9%**	**84.4%**
BP controlled in diabetes	2011	82.3%	**83.7%**	83.0%
Diabetic nephropathy	2011	31.5%	30.5%	32.1%
Cholesterol tested, 35-54y	2003	60.2%	**86.1%**	**88.1%**
Cholesterol tested, 55-74y	2003	63.2%	**76.7%**	75.3%
LDL controlled	2013	83.5%	**83.9%**	84.2%
LDL < 100 in high risk patients	2013	27.8%	**30.8%**	34.4%
BP documented, 20-54y	2003	21.8%	**91.2%**	**92.5%**
BP documented, 55-74y	2003	30.6%	**81.9%**	80.8%
Body-mass index documented	2008	56.5%	**88.2%**	**89.6%**
Smoking status documented	2011	79.4%	**87.9%**	**90.7%**
Statins in ischemic heart disease	2014		82.2%	82.4%
Hemoglobin test in infants	2008	71.7%	**85.3%**	**88.3%**
Anemia in infants	2014		8.1%	8.2%
Benzodiazepines, elderly	2011	4.9%	5.2%	4.8%
Tertiary prevention, hip fracture	2015	25.5%	25.5%	28.1%
Antibiotics DDD per 1000	2014		20.8	19.1
Use of cephalosporins/quinolones	2014		22.1%	25.2%
Community psychiatrist care following psychiatric admission	2015		32.5%	37.3%

Bold print represents substantial improvement in comparison with previous time point (see Methods section). *COPD* chronic obstructive pulmonary disease, *PAP smear* Papanicolaou smear, *LDL* low-density lipoprotein, *BP* blood pressure, *DDD* defined daily dose

Health plans also maintain internal data on regions, clinics, and individual physicians. The plans and their clinicians have made intensive use of these data to bring about substantial improvements in quality [28]. Clinics are held accountable through extensive data collection and management of their performance by health plans. Data are based on the electronic patient records designed as well to monitor the quality of health services delivered through its four competing health plans [10].

According to Elhayani [28], data regarding a clinic’s performance in quality is used as “a major information tool” for planning the clinic’s quality improvement activities. This is done by (1) identifying clinical domains in which the clinic’s performance is markedly different from that of other clinics; (2) establishing an annual work plan; (3) doing on-going performance monitoring, including assessing comparative performance of clinicians within the clinic. Medical teams seem to be eager at getting the information. Reflecting these data to clinics encourages self-improvement processes and awareness. Information is used in professional team meetings and increases the uptake of clinical guidelines in clinics. The staff’s adherence to their own protocols is often higher than compliance with national and international guidelines [28].

In a comparative study of national quality improvement systems from seven countries, the Israeli program stands out in several aspects: (1) it is operated by the academy rather than by the government; (2) its core aim is not public reporting but rather to provide information on healthcare quality at the national level; (3) it uses quality indicators only, as compared with some countries which use other tools as well, such as site visits, national accreditation, qualitative standards, and peer reviews; (4) it performs extensive risk adjustment; (5) it relies exclusively on data from electronic medical records; (6) it does not present data on a regional level; and finally (6) there are no consequences for performing below expectations [29]. Public reporting would be an important way of bringing the public into either demanding more or faster quality improvement efforts of their health plan or do it by increasing competitive pressure on the health plans should there be significant movement of people from one plan to another as a result of their interpretation of the data. While risk adjustment helps in overall assessment of performance by health plans, it obscures differences in a plan’s performance for certain groups of people. Looking at stratified data vs. adjusted data is an important way of seeing such disparities. Another aspect would be the presentation of data by regions, with expected performance correlated with other resources which are not equally distributed. Finally, some penalties for under-performing could move the performance rates into a higher level.

Until recently, patients in Israel had little basis on which to make informed choices between health plans, should they wish to do so. The issue of choosing providers within health plans or choice of hospitals for ambulatory services is even more complicated, due to the limited choice for patients allowed by health plans. According to the OECD report [9] “Many within the Israeli health system have argued that publishing quality of care indicators would lead to consumers making skewed assessments of performance, as these indicators do not provide holistic measures of good quality healthcare” [9]. However, this is not more than a symptom of resistance to transparency. If the consumers seem to be making a skewed assessment, it might be claimed that it is up to the health plans to help them make a better assessment by providing even more information.

The OECD report concluded that “too little is known about the quality of care delivered in hospitals.” This lack of information was described as “particularly concerning”. The authors added that “Hospitals should have access to data on how they compare on quality measures … that can be used to inform improvements in care … the development of a national dataset that allows hospitals and plans to compare their performance relative to their peers, remains in its infancy. The government’s efforts on this front ought to be more ambitious and rolled out more quickly” [9].

At the next stage, the National Quality Indicators Program for Hospitals was launched in 2012. The program, led by the MOH, assesses care of all general, psychiatric, and geriatric hospitals and also mother and child clinics and pre-hospital emergency care. This program is regulatory-based and the institutions involved are required to report administrative and clinical data relevant to quality indicators selected by a mostly academic steering committee. Annual public reports have been issued since 2015. As of 2020, the program includes 65 quality indicators [3, 30, 31]. In 2020, one indicator related to brain ultrasound in neonates was introduced, and another one, related to polyp detection rate in screening colonoscopy, is to be added in 2021 [31]. Of 16 indicators listed in Table 2, most (14 indicators, 88%) show substantial improvement over time, but several indicators have much room for improvement, including median time to triage in the emergency department and repeated visits to the emergency department.

**Table 2:** Performance in quality indicators in general hospitals over time [30]

Indicator	First Year	Performance in the first year	Performance, 2018
Primary coronary intervention within 90 minutrs in STEMI	2013	68%	**91%**
Statins in acute coronary syndrome	2017	90%	**93%**
Median time (minutes) to CT/MRI in suspected stroke	2015	55	**29**
Carotid artery imaging in transient ischemic attacks	2015	58%	**83%**
Hip fracture surgery within 48 h	2013	71%	**87%**
Prophylactic antibiotics in colorectal surgery	2016	78%	**85%**
Prophylactic antibiotics in hip fracture surgery	2014	66%	**88%**
Prophylactic antibiotics in cesarean section	2014	78%	**95%**
Risk assessment for venous thromboembolism	2014	62%	**95%**
Anticoagulants in women undergoing hysterectomy	2014	57%	**96%**
Pain control in the post-operative care unit	2016	86%	**97%**
Avoiding hypothermia in the post-operative care unit	2017	78%	**91%**
Median time (minutes) to triage in the emergency department	2017	10	10
Readmission to the emergency department	2015	5.6%	5.4%
Pre-partum steroids for women in risk of preterm delivery	2016	95%	**98%**
Avoiding hypothermia in neonates	2017	55%	**71%**

Bold print represents substantial improvement in comparison with previous time point (see Methods section)

*STEMI* ST-elevation myocardial infarction, *CT* computerized tomography, *MRI* magnetic resonance imaging

It should be noted that the National Program for Quality Indicators in Hospitals publishes targets for each indicator, including whether each hospital met or did not meet the set goal. In its early phase, performances above the target were reported, so that over-competition between hospitals actually led to the target being perceived as 100%. This led to criticism, specifically, for the indicator of performing hip fracture surgery within 48 h. From a clinical perspective, it is not always clear that early hip fracture repair surgeries should be encouraged, as some patients may be unsuited for immediate surgery. If these patients are rushed to surgery, they will not always benefit from the intended clinical effects; in fact they may be at a higher risk for adverse outcomes. As the organizational logic of following requirements to meet the established quality parameter pushes practitioners towards maximization of early surgeries (with the parameter being publicly compared between hospitals in the media), there is a danger of contraindicated patients being operated on anyway, thus compromising their survival. This example also highlights that reliance on a single parameter creates a risk of this quality measure not being exhaustive enough, i.e. not including the whole spectrum of factors required for proper assessment. In order to minimize this risk, quality measures should be adjusted for other considerations, e.g. co-morbidity or chronic medications [32].

In addition to the national quality indicators, some hospitals develop their own internal quality indicators. For example, Clalit owns 14 hospitals; and it has developed internal quality indicators for them as of 2005. Furthermore, each hospital develops internal quality indicators for its own internal benchmarking and improvement [33].

Another important development is the initiation of quality indicators for the continuity of care between hospitals and the community, e.g. time from performing a mammography which raises the suspicion of a malignant breast lesion to the initiation of surgical or oncological care for women with breast cancer [33, 34]. These efforts are still anecdotal and have not matured into system-wide measurement. There are other indicators of continuity that are important from the perspective of patients, e.g., is pertinent information being transmitted to their primary care practice about their visit in the emergency department; or when the patient is admitted to the hospital, is this information known immediately to the patient’s primary care practice? All of the health plans and hospitals now use electronic medical records. Israel has also developed an information highway [35] that facilitates transfer of information between the hospital and ambulatory sectors of the health system. These electronic tools facilitate the transfer of the information needed to facilitate continuity of care; but the use of the tools for this purpose is not being monitored at this time. Moreover, information is not consistently located in the same place, e.g. chronic medications and underlying diagnoses are presented in a more convenient way for enrolees of some of the health plans and less conveniently for enrolees of other health plans. The increased use of electronic medical records in health plans and hospitals and the widespread use of the information highway have facilitated the transfer of such information; but its actual use is not being monitored at this time.

### Minimizing heath disparities

In Israel, disparities between population groups have been demonstrated for decades. Israel is a country with higher poverty and income inequality rates than most other OECD countries, and persistent disparities in health have been documented along the socio-economic gradient, and with regard to ethnicity, country of birth, religion, gender, and geographic location within Israel. These disparities include health outcomes, disease risk factors, mortality rates and self-rated health [36, 37].

In 2010, the Israel MOH made the reduction of health disparities as one of its strategic objectives. This national plan for tackling health inequalities is focused primarily on impacting midstream factors (e.g., with in the healthcare system, which are under its direct purview), such as improving access to critical healthcare-service infrastructures in peripheral areas; eliminating financial and other access barriers to care for low-SES population groups; reducing disparities in access to and quality of healthcare services due to cultural barriers; developing incentives and tools that support the efforts of ‘agents of change’ in combating health disparities among target groups, and establishing a national health disparities database. Reporting on and monitoring of health disparities was an integral component of this new policy [37]. The MOH publishes a comprehensive annual report dedicated solely to this subject, which details specific programmes by each health plan and other groups for addressing health disparities [38, 39]. The National Program for Quality Indicators in the Community [20] also includes data stratified by socioeconomic status, which allows estimating gaps in quality. The above-mentioned OECD report commented that “Israel’s health system has to contend with a complex picture of health inequalities. In general, those who are not Jewish, live in the North or South, and those from other poor socio-economic groups are likely to suffer from poorer health outcomes” [9].

Efforts have been undertaken to overcome cultural factors and language barriers that often limit disadvantaged groups from getting the most out of health services, including efforts to direct health plans and providers to deliver information and advice in multiple languages [9]. In addition to the MOH health disparity reports, a health disparities knowledge center was established that publishes additional data on health disparities based on in-depth data analyses of the Central Bureau of Statistics’ surveys as well as original research [32]. Recently, the National Institute for Health Research Policy funded a study aimed at developing a set of measures for monitoring and public reporting of gaps in healthcare. MOH activities aimed at reducing health disparities include compiling, analysing, and publicly disseminating information about health care disparities, including periodic reporting of variations in health and health care access and instituting an annual conference showcasing initiatives to reduce disparities [37].

Clalit health plan has its own strategy aimed at reducing health disparities. The two main components of the strategy are (1) a top-down approach of disparity-reduction goal setting, continuous monitoring, and provision of incentives; and (2) use of tailored interventions to reduce disparities by a bottom-up approach in which each district and locality is empowered to plan interventions, policy changes, and shifts in workforce. The strategy involves the following main steps: selecting quality indicators, rating the primary care clinics, selecting target clinics for intervention, setting goals, providing feedback and support to district steering committees for suggested interventions, continuous monitoring, and incentives [40, 41]. Target clinics implemented on average 17.5 interventions per clinic. Interventions focused on decision support and Community linkages were positively correlated with improvement in the composite quality score. Conversely, focusing on a specific clinical domain was not correlated with a higher quality score [42]. In a further study of this intervention [43] it was found that successful clinics were those that had a either (1) highly effective teamwork, a small gap to minimize, and employing a wide range of interventions; (2) large gap to minimize with high clinic team work, focusing efforts on tailoring services to their enrolees; or (3) clinics having medium to low teamwork but strong middle-management support [43].

The Advisory Committee for Strengthening the Public Health System in Israel recognized waiting times for medical services as a major problem. The committee made several recommendations aimed at reducing waiting times in the public system. These included setting legal standards regarding geographic access and waiting times as well as establishing a national monitoring center for waiting times, and publishing periodic reports on waiting times by procedure, hospital and health plan [44, 45]. Such a system has been recently implemented, when the MOH launched a GIS-based system for demonstrating waiting time for five disciplines in primary care (dermatology, ENT, ophthalmology, OB/GYN and orthopaedics) [44]. However, this system is limited to the public sector, while going to a private physician is another way of getting around waiting. Thus, actual waiting times are not monitored and data regarding access to private physicians in rich people vs. poorer people is not available. Indeed, a recent report showed that Arabs, chronically ill patients and people living in poverty were less likely to have private healthcare insurance [38].

Other activities aimed at reducing healthcare disparities included legislation, training staff for cultural competence, initiating an interpretation phone line for immigrants from Ethiopia, financial incentive for health plans to reduce disparities, investing money in building and renovation of clinics and hospital in the periphery [38]. Many independent programs, initiated by the various health plans, have aimed to reduce inequalities in health by a combination of policies. These include lowering co-payments, focusing on at-risk populations, and adapting intervention measures to language, culture, literacy, and comprehension levels [38].

Other health plans also report focused efforts for decreasing healthcare disparities: Maccabi Health Services developed specific intervention for poor elderly people, interventions for health diet and physical exercise among Bedouin Arabs and Arabs in East Jerusalem, controlling diabetes in Bedouin Arabs and Arabs in Jaffa and the Sharon area, health promotion among the ultra-orthodox citizens of Bnei-Brak and in young Arab families, increasing access to care for Maccabi enrolees in Northern Israel. Meuhedet focuses on elderly poor individuals, child development, and empowering women in minority groups (ultra-orthodox Jews and Arab Bedouins)**.** Leumit has a program to reduce racism, improve accessibility and focused interventions for controlling diabetes in Ethiopian Jewish immigrants, and increasing the adherence to vaccines in Arab and Ultra-orthodox populations. All health plans promote increased access to care among patients with disabilities [38].

Table 3 demonstrates trends in two aspects of health disparities: those between Jews and Arabs and those between Central Israel and its periphery (i.e. the Northern and Southern districts). As far as ethnicity is concerned, it can be shown that little progress has been made, as only 4 of 11 indicators (36%) show substantial improvement over time in term of closing the gap. When looking at geography, 7 of 16 indicators (44%) show substantial improvement, although most of the closing of the gap stems from resources in Central Israel becoming more scarce and similar to those of the periphery, rather than the other way around [37, 38].

**Table 3:** Selected statistics showing inequality in healthcare parameters by ethnicity and geographic location [38, 39]

Indicator	First Year	Difference in the First Year	Last Year	Difference in the Last Year
**Differences between Jews and Arabs**				
Life expectancy at birth, males	1975	3	2018	3.7
Life expectancy at birth, females	1975	3	2018	2.8
Infant mortality / 1000 live births	1975	−23	2017	**−2.5**
Age-adjusted cancer / 100,000, males	1974	110	2015	**45**
Age-adjusted cancer / 100,000, females	1974	170	2015	100
First visit to maternity care	2016	10%	2018	14%
Exclusive breastfeeding at birth	2016	17%	2018	**16%**
Complementary insurance ownership	1997	32%	2017	45%
Private insurance ownership	1997	15%	2017	35%
Influenza vaccine	2010	8%	2017	**2%**
Mammography screening	2010	15%	2017	16%
**Differences between center and periphery**				
Infant mortality / 1000 live births	2008	−1.9	2017	−1.7
Prophylactic antibiotics, hip fracture surgery	2014	26%	2018	**13%**
Evaluation for violence in psychiatric ED	2014	−12%	2018	5%
General hospital beds / 1000	2008	0.75	2018	0.69
Internal medicine beds / 1000 > 45y	2008	0.65	2018	0.53
ICU beds / 1000	2008	0.04	2018	**0.02**
Pediatric beds / 1000 0-14y	2008	0.38	2018	0.41
Surgical beds / 1000	2008	0.45	2018	**0.32**
Obstetric beds / 1000	2008	0.17	2018	**0.01**
ED positions / 1000	2008	0.08	2018	**0.03**
Surgical suits / 1000	2008	0.04	2018	**0.03**
Post-operative care beds / 1000	2008	0.08	2018	**0.04**
Israel graduates among physicians	2008	13%	2020	15%
Physicians / 1000	2011	1.4	2016	2.0
Nurses / 1000	2011	1.6	2016	1.9
Allied health professionals / 1000	2011	1.5	2016	1.4

Bold print represents substantial improvement in comparison with previous time point (see Methods section)

*ED* emergency department

### Accreditation

Accreditation by Joint Commission International (JCI) was first introduced to hospitals owned by Clalit in 2006. The process started with an educational workshop to hospital leaders held in 2005 and followed by establishing a steering committee which included representatives from all participating hospitals and senior managers at Clalit headquarters. A dedicated website was built on Clalit’s intranet, and mock surveys were performed in each hospital introduced into the accreditation process. Eleven committees with 150 representatives worked for 1 year mapping gaps between JCI standards and existing policies [46]. Dozens of new policies were written during the following 15 years. Later, “peer mock surveys” were introduced into Clalit, where colleagues from some hospitals and Clalit perform a condensed mock survey similar to the one performed by JCI, with a full exit report submitted to the hospital leadership a few weeks later.

MOH’s Medicine Division directive 38/2012 [14] states that as of July 2015, all general hospitals will be required to undergo accreditation by JCI “or be in the process of receiving JCI accreditation” as an obligatory condition for licensing. More and more Israeli hospitals are undergoing the JCI accreditation processes, which might contribute to significant improvements in quality of care and safety. In addition, the MOH has recently begun to increase the intensity with which it monitors and promotes the quality of hospital care [2]. As of May 2020, 29 Israeli hospitals have undergone JCI accreditation, as listed on the JCI website [47]. Since the reports issued by the JCI are confidential, there are no data available to demonstrate an improvement over time, e.g. a decrease in the number and / or severity of survey findings over time. At the hospital level, a key goal of the accreditation process is to create an improvement-oriented climate, to encourage teamwork and quality improvement, and to foster staff’s technical and mental preparedness for essential organizational changes [48].

### Financial incentives

There are currently no explicit financial incentives for hospitals and health plans to improve quality. However, owing to the competitive environment, public dissemination of quality data may be providing an indirect incentive [28]. In recent years, several pay-for-performance incentives have been introduced by the MOH. These deal with patient safety, prevention and control of infections, as well as the quality of care in the emergency department and the neonatal care unit. Comparative data are available for some of these programs. The pay-for-performance program for neonatal care units, started in 2014, has led to a 76% decrease in hospital-acquired infections, an increase in 102 physicians (78 residents and 24 specialist), 396 nurses, advanced training in neonatal intensive care for over 300 nurses, additional 20 infection prevention and control nurses and 159 allied health professionals (pharmacists, physical therapists, psychologists, social workers, respiratory technologists, dietitians, occupational therapists, speech therapists), as well as improvements in infrastructure. Overall performance on a set of neonatal care indicators increased almost three-fold between 2014 and 2019, from a score of 33 up to 92 [49]. A similar program for improving infection prevention and control [50] is described in the next section.

### Infection prevention and control

Since the 1980s, Israel has already had the basis for prevention and control of health-associated infections (HAI) by means of professionals working in this field, which formed associations, promoting events of scientific updating and periodic debates around this issue. Some professionals had specific training in HAI prevention and control in institutions outside Israel. The national Israeli program for infection control and antimicrobial resistance was established in 2007. Some of the actions of this unit over the years included publishing guidelines on prevention and control of HAI, establishment of a surveillance system; regulations on infection control committees in hospitals and supervision of infection prevention practices by means of systematic visiting to health facilities The program staff reviews their guidelines and publishes their results periodically [51]. MOH regulation [52] defines the structure needed for effective prevention and control of HAI. This structure includes the National Centre for Infection Control and Avoiding Antimicrobial Resistance as part of the MOH. Every hospital with more than 100 beds is required to have an infection prevention and control program. The structure of this unit depends on the size of the hospital. Hospitals with more than 400 beds are required to have a designated unit for infection prevention and control headed by a physician who is board-certified in infectious diseases or clinical microbiology and has experience in infection prevention. The unit should include one full-time equivalent nurse for every 200 beds, a data and statistics analyst, and supported by a clinical microbiologist, pharmacists and secretary services. Some of the roles of this unit include monitoring and surveillance of HAI, monitoring the use of antibiotics, consulting to leadership and staff regarding the use of immunizations, investigation of outbreaks and reporting to the hospital leadership and the MOH. Another requirement is to establish a multidisciplinary committee for infection prevention which convenes at least four times a year [52].

At the hospital level, strategies used for prevention and control of hospital-acquired infections include implementing promoting hand hygiene, antibiotic stewardship, cohorting of patients with antibiotic-resistant bacteria, disinfection and sterilization of medical equipment, monitoring the quality of cleaning of the patient’s unit, implementing standards of infection prevention into the electronic medical record, and restricting the use of broad-spectrum antibiotics [33]. As mentioned above, a pay-for-performance program has been initiated in recent years [50]. This program reported a decrease in hospital-acquired infections over several years, which ranged from 27% decrease in *Clostridium difficile* infections to a 70% decrease in central-line associated blood stream infections. Overall, there was a 45% improvement in the mean score from 2018 to 2019 [50].

Improvements in infection prevention are listed in Table 4. Major improvements in these objective indicators, most of which are outcome measures, can be demonstrated (10 of 13 indicators, 77%, have improved substantially). Challenges remain in reducing the incidence of catheter-associated urinary tract infection and introducing antibiotic stewardship into primary care. In addition, data regarding acquired infections with certain resistant bacteria, such as methicillin-resistant *Staphylococcus aureus* and vancomycin-resistant *Enterococcus,* are published only as number of cases and not incidence rates, and are therefore not included in Table 4.

**Table 4:** Infection prevention and control indicators over time [53–57]

Indicator	First Year	Incidence in the First Year	Last Year	Incidence in the Last Year
CPE, long-term geriatric centers /100,000 patient-days	2010	32	2019	**4**
CPE, nursing homes / 100,000 catheter-days	2010	7.8	2019	**0.8**
CLABSI, all ICU types / 1000 catheter-days	2012	7.5	2019	**2.1**
*C. diff*, general hospital beds / 100,000 patient-days	2016	35.6	2018	**31.5**
*C. dif*f, non-general hospital beds / 100,000 patient-days	2016	24.4	2018	**14.4**
CAUTI, internal medicine / 1000 catheter-days	2017	3.6	2019	**3**
CAUTI, surgery/1000 catheter-days	2017	2.5	2019	3
CAUTI, geriatrics/1000 catheter-days	1997	2.8	2017	4
Antibiotic use, intensive-care unit, DDD / 100 patient-days	2012	131.7	2018	**113.1**
Antibiotic use, internal medicine, DDD / 100 patient-days	2012	89.9	2018	**75.1**
Antibiotic use, surgery, DDD / 100 patient-days	2012	94.7	2018	**79.9**
Antibiotic use, long-term geriatric centers, DDD / 100 patient-days	2012	26	2018	**14**
Antibiotic use, primary care, DDD / 1000 enrollees/day	2012	18.5	2018	17.8

Bold print represents substantial improvement in comparison with previous time point (see Methods section)

*CPE* Carbapenemase-producing *Enterobacteriaceae*, *CLABSI* centeral-line associated blood-stream infection, *C, diff Clostridium difficile*, *CAUTI* catheter-associated urinary tract infection, *DDD* defined daily dose

In the community level, antibiotic stewardship remains a challenge, with a recent study [57a] showing increased use of antiobiotics in primary care (20.7 defined daily doses per 1000 per day, similar to the OECD average, with 22% second-line antibiotics (higher than the OECD average of 17% [58].

### Patient safety

Some strategies used for improving patient safety in the Israeli healthcare system include mediation between the patient and the system (a process where nurses, ombudsmen and staff from the risk management unit talks to patients who have complaints regarding the quality of care and tries to avoid a malpractice suit by bridging between conflicting views); orientation days and updates to staff regarding a variety of topics, including patient safety and quality of care, spreading knowledge via email regarding adverse events that occurred and what can be learned from them, safety rounds in departments where the ward leaders talk about their concerns for quality and patient safety, often led by the director general of the hospital and the nursing director. Another important topic is implementing a culture of safety in hospitals [33]. A survey conducted by the MOH in 2012–2015 showed that the safety culture in hospitals accredited by JCI was better than in those hospitals which did not undergo accreditation [33, 59]. The Patient’s Rights Law (1996) defines a Review and Quality Committee which looks at medical procedures and processes from a wide perspective and allows in-depth review quality improvement of these processes under legal privilege. The confidentiality of these reports facilitates openness of medical teams and discussion of sensitive issues, root cause analysis and suggesting recommendations for improvement. In 2001, when this tool was seldom used, it was described as “disruptive to quality improvement efforts” [4]. However, in recent years these committees gain increasing popularity at all levels of the system, from the MOH and health plans to individual hospitals, while an opportunity to increase their widespread use still exists.

The patient safety unit in the MOH publishes guidelines and reports. As mentioned above, a pay-for-performance program has been initiated in recent years. This patient safety initiative deals with patient identification, work processes in the operating room, training in patient safety for staff, implementing safety rounds, quality improvement projects at the department level, reporting of near-miss events and learning from adverse events [60].

Some indicators taken from the Patient Safety pay-for-performance program are shown in Table 5. Over the course of 2 years there has been some modest improvement (5 of 8 indicators, 63%, have improved), though this program awaits further growth and development.

**Table 5:** Indicators of patient safety over time [60]

Indicator	Performance, 2017	Performance, 2019
Patient identification in the ED	79%	**80%**
Patient identification in the imaging department	83%	**86%**
Pre-operative data verification	93%	92%
Counting of items during and at conclusion of surgery	95%	85%
Training staff in patient safety	65%	**89%**
Performing safety round according to MOH methodology	57%	**89%**
Performing quality improvement projects using PDSA methodology	82%	**96%**
Performing root-cause analysis of sentinel events using 5 M methodology	96%	100%

Bold print represents substantial improvement in comparison with previous time point (see Methods section)

*ED* emergency department, *MOH* Ministry of Health, *PDSA* Plan-do-study-act

### Improving patients’ experience

Improving patient experience is a major strategic goal for the MOH and for health plans. A specific department at the MOH deals with patient’s experience, and its goal are to set standards for patient experience and service, building assessment tools and to set the national policy for improving patient experience [61]. Four aims were set by the MOH: (1) to define policy and lead processes to improve the patient experience and quality of service to patients in the healthcare system; (2) to implement values of service and patient-centred organizational culture; (3) to define measures for patient satisfaction and patient experience, monitor and publish them; (4) to encourage healthcare organizations to set patient experience as a strategic goal. A practical guide for health organizations was published by the MOH in 2016 [61]. This guide gives “tips” on dealing with difficult situations in the patient’s journey in various healthcare settings. In addition, it lists several mechanisms that should be used in an organizational perspective to improve patient experience. These include appointing a person in charge of patient experience in the organization, mapping the patient’s journey, establishing focus groups with patients, observations, surveys, in-depth interviews with patients and providers, integration patients and families in improvement processes, defining goals and values, as well as standards for patient experience, incorporating patient experience into annual work plans at the unit level, and conducting surveys at least annually [61].

In 2014, the MOH published the results of nationwide surveys of hospitalized patients regarding their satisfaction and similar surveys have been carried out in 2016 and in 2018. Similarly, patient satisfaction in the emergency department is also monitored since 2017. The MOH has also assembled a database of waiting times for surgical operations, with the intention of publishing updated comparative data in the near future [2]. The Myers-JDC-Brookdale Institute conducts a yearly survey of patient satisfaction with the primary healthcare system. The recent survey, conducted in 2019, included 3508 respondents [62]. Tools used for improving patient experience by Israeli hospitals include internal surveys, focus groups with patients, “round tables” with employees, training on patient experience for employees, and establishing an institutional Service and Patient Experience Unit which manages all these activities, hires external consultants and guide department in their efforts for improving patient experience [33].

Surveys of patient satisfaction in the community have been conducted over the years by JDC-Brookdale [62–64] and show some improvement over time (Table 6]. Similar surveys were conducted in the emergency department [65] and in-patients [66] and similarly show substantial improvement in patient satisfaction in most indicators (Table 6).

**Table 6:** Indicators of patient satisfaction over time [62–66]

Indicator	First Year	Performance in the First Year	Last Year	Performance in the Last Year
**Patient satisfaction with the primary care system**				
General satisfaction with the health plan	1995	83%	2018	90%
Satisfied with the primary care physician’s attitude	1995	89%	2016	**94%**
Satisfied with the primary care physician’s professionalism	1995	81%	2016	**91%**
Satisfied with the primary care nurse’s attitude	1995	86%	2016	**96%**
Satisfied with consulting physicians’ professionalism	1995	78%	2016	86%
Satisfied with the administrative staff’s attitude	1995	81%	2018	86%
Satisfied with laboratory services	2016	78%	2018	**95%**
**Patient satisfaction with the emergency department**				
Overall score	2015	66%	2019	**74%**
General satisfaction	2015	56%	2019	**65%**
Felt to be “in good hands”	2015	69%	2019	**76%**
Would recommend the hospital	2015	59%	2019	**67%**
Respect and dignity	2015	75%	2019	**83%**
Information and explanations	2015	69%	2019	**78%**
Continuity of care	2015	67%	2019	**76%**
Perceived waiting time	2015	67%	2019	**73%**
Physical conditions	2015	66%	2019	**75%**
**Patient satisfaction with in-patient services**				
Overall score	2014	77%	2018	**81%**
General satisfaction	2014	75%	2018	77%
Felt to be “in good hands”	2014	84%	2018	**88%**
Would recommend the hospital	2014	74%	2018	75%
Respect adignity	2014	83%	2018	**88%**
Information and explanations	2014	76%	2018	**84%**
Continuity of care	2014	77%	2018	**82%**
Efficiency	2014	79%	2018	**84%**
Physical conditions	2014	76%	2018	**78%**
Patient’s empowerment	2014	79%	2018	**84%**

Bold print represents substantial improvement in comparison with previous time point (see Methods section)

### Training for quality

Courses in quality and patient safety are gaining increasing popularity in medical schools and Master of Health Administration programs. Research of clinical quality is also on the rise [3]. In 2012, the OECD wrote that hospitals should be encouraged “to develop their own programs to foster a culture of quality improvement amongst their staff.” [9].

### Additional approaches

Israel has adopted a number of programs and methods used in other developed countries for improving and ensuring quality of care. These include a variety of health information technology, such as electronic medical records, the Picture Archiving and Communication System (PACS) and the “Choosing Wisely” initiative to eliminate unnecessary care [33].

### Challenges and barriers to quality improvement efforts

Several barriers for widespread use of quality improvement methods in the Israeli healthcare system include reluctance by unions, such as the Israel Medical Association and the recent struggle of the National Association of Nurses in Israel against JCI accreditation, the partial, inconsistent and unreliable flow of information in the system, and the failure to incorporate quality improvement initiatives and / or monitoring as a criteria for relicensing of hospitals and recognition of departments as eligible for residency by the scientific council of the Israeli Medical Association [4]. When reviewing the literature, reasons cited for objection to quality indicator programs cited include lack of agreement with the measures, concerns that such programs focus attention on what can be measured at the expense of more important aspects of care that cannot be measured, concerns about unintended consequences, and insufficient adjustments for factors thought largely to be beyond the control of individual physicians, such as the case mix of their population or patient adherence to recommendations. It was also claimed that such systems lead to improvements in documentation, rather than fundamental improvements in quality of care. Finally, many physicians believe that such systems may be an affront to their notions of autonomy [67]. A survey of primary care physicians [68], demonstrated broad support for the quality measurement program. Almost all physicians felt the program was important or very important and supported the continuation of the program. Even more importantly, half of physicians had made changes in their practices to improve performance. There was widespread support of the monitoring program’s choice of broad clinical areas. There was also a fair amount of support for the way the specific indictors had been defined. Physicians reported several problems that they associated with the program: increased workload, over-competition, excessive managerial pressure, distraction from other clinical issues, concerns about the validity of some of the quality standards, encouragement of unnecessary tests or treatments, and decreased autonomy so they had limited ability to deviate from the protocols embedded in the quality indicators in those cases where it made sense clinically to do so [68]. Levi [69] claims that “Some critics … point to the difficulties and dangers in quality measurement, especially when it is connected to financial incentives or to the comparative publication of results of care providers.” The use of tools of supervision, evaluation, monitoring and measurement, ultimately diminishes doctors’ professional autonomy. According to this perception, professional autonomy itself depends on the inability of the lay-man to properly evaluate, assess or appreciate the esoteric work of the expert, making medical professionals the only authority able to judge the quality of their own work. The potential threat to medical dominance stems from quality measurement on the one hand, and the growing ambition of the regulatory state to apply rigid measurement mechanisms to healthcare services on the other hand. Information based on measurement offers both managers and the public the ability to judge medical performance. However, it holds the potential of undermining medical authority. The National Program for Quality Indicators in Hospitals “does not appear to have aroused opposition from the Israel Medical Association” [69]. Similarly, family physicians’ support for the National Program for Quality Indicators in the Community “remained high over the years despite specific and anecdotal criticisms regarding several indictors, their interpretation and occasionally over managerial demands developed in response to unsatisfactory outcomes” [69]. A more radical view is brought by Fisher [70] who claims that quality measures have negative impact and could “damage the implementation of a measures-oriented medical policy, dictated and controlled by management”. Some physicians are “fed up with instruction on managing quality in medicine and feel that they serve the interests of their managers more than their patients”. They claim that physicians are not interested in the competition associated with quality measurement. They claim that quality measures brought top-down do not allow for the individual and creative selection of quality improvement initiatives by healthcare professionals. Another problem mentioned in this paper is “gaming” of data, even to the point of falsifying medical records for the sake of meeting measures’ goals [70]. Thus, the reliability and validity of the data used for assessing indicators’ performance can be compromised. Another problem is the cross -sectional nature of “snap shots” used for creating the National Program reports, rather than a longitudinal one. The latter can produce important information missing from the former. For example, in study by Paltiel et al. [71] it was shown that while the adherence to colorectal screening in cross-sectional reports from the National Program for Quality Indicators in the Community reached 65% in 2012, only 25.5% of the population demonstrated full longitudinal screening adherence, mainly attributable to colonoscopy in the past 10 years rather than annual fecal occult blood test performance. Another often cited problem is “tunnel vision”, where managerial focus is given to areas being measured, not necessarily to the most important aspects. The problem of a “ceiling effect” where setting a less-than-optimal goal for an indicator causes providers to align their improvement effort when they reach the target has also been described. The usage of quality indicators to assess socio-economic inequalities, as well as changes in inequalities over time necessitates appropriate data on socio-economic status, yet direct data at the individual level on socio-economic status is a challenge within the electronic medical record systems, both for health plans and for hospitals. Most quality indicators used by these programs are process indicators, while outcome indicators are rarely used.

Tal [72] lists several limitations of hospital accreditation. These include the challenge of adjusting the process to local characteristics of the healthcare system, its high cost, the limited evidence of its impact on quality. Tal claims that national programs for quality measurement or for promoting patient safety are more cost effective.

The MOH lists several barriers to improving patient experience. These include awareness by managers and healthcare providers, lack of resources (understaffing, overload), burnout and compassion fatigue, gaps in provider-patient perception and communication, challenges in physical infrastructure, methodological issues related to measuring of patient experience and bureaucracy [61].

### Concluding comments

The Israeli healthcare system has adopted several mechanisms for promoting quality and patient safety at various levels. These include legislation, financial incentives, quality indicators, patient experience, prevention and control of infection, accreditation, and the widespread use of electronic medical records. Several aspects, such as peer review, the “choosing wisely” initiative, training for quality, and minimizing health disparities, could be implemented more intensively and effectively. In addition, there is heterogeneity in outcomes: Quality indicators, infection control and patient experience in primary care and the emergency department have all shown substantial improvement; Minimizing disparities, achieving greater improvements in patient safety and in patient experience for inpatients, remain challenges.

Several stakeholders in Israeli healthcare system can do much more than is currently done to improve quality. While we do not recommend establishment of a “Quality Institute” [4] we think that the MOH could form an alliance with the Israeli Medical Association and the National Association of Nurses in Israel to introduce peer review similar to that of the European Foundation for Quality Management. Endorsing an Israeli “core curriculum” for quality improvement and patient safety, to be used at all levels (from medical and nursing schools to residency programs and continuous medical education, by the MOH and the Scientific Council of the IMA) could improve the knowledge base on this topic. Reducing healthcare disparities requires additional budgets from the MOH, as well as support from health plans. This could be promoted by introducing strong financial incentives to minimize gaps.

Overall, the Israeli government and health system are working to improve quality of care in multiple ways, have good data to understand what is working and what needs particular attention, and have the tools to continue to improve.

## Data Availability

Not applicable.

## References

[1] van der Zee J, Kroneman MW (2007). Bismarck or Beveridge: a beauty contest between dinosaurs. BMC Health Serv Res.

[2] Rosen B, Waitzberg R, Merkur S (2015). Israel – health system review. Health Syst Transit.

[3] Clarfield AM, Manor O, Bin-Nun G, Shvarts S, Azzam ZS, Afek A, Basis F, Israeli A (2017). Health and health care in Israel: an introduction. Lancet..

[4] Porath A (2001). Quality in the Healthcare System at the Age of Modernization.

[5] Ministry of Health (2018). Burnout Survey Results.

[6] Nolte E, McKee M (2004). Does healthcare save lives? Avoidable mortality revisited.

[7] Goldberger N, Haklai Z (2012). Mortality rates in Israel from causes amenable to health care, regional and international comparison. Isr J Health Policy Res.

[8] McKee M, Chow CK (2012). Improving health outcomes: innovation, coverage, quality and adherence. Isr J Health Policy Res.

[9] OECD. OECD Reviews of Health Care Quality: Israel. 2012. http://www.oecd.org/israel/ReviewofHealthCareQualityISRAEL_ExecutiveSummary.pdf. Accessed 2 May 2020.

[10] Blum N, Halperin D, Masharawi Y (2014). Ambulatory and hospital-based quality improvement methods in Israel. Health Serv Insights.

[11] Israel Health Policy Research Institute. 19th Dead Sea Conference - 25 years to the National Health Insurance Law: challenges and modifications needed for the coming decade”, 2019. http://israelhpr.org.il/publications/.

[12] Ministry of Health. National Health Insurance Regulations: Quality Indicators and Data Disclosure. Jerusalem: Ministry of Health; 2012 [Hebrew].

[13] Ministry of Health, Medicine Division directive 22/2014, The National Program for Quality Indicators in Hospitals: Monitoring and Follow-up. Jerusalem: Ministry of Health; 2014 [Hebrew].

[14] Ministry of Health, Medicine Division directive 38/2012, Accreditation / Certification Process in General Hospitals. Jerusalem: Ministry of Health; 2012 [Hebrew].

[15] Ministry of Health, Medicine Division directive 35/2012, The Patient Safety Unit in Hospitals: Structure and Roles. Jerusalem: Ministry of Health; 2012 [Hebrew].

[16] Ministry of Health, Medicine Division directive 48/2013, The Patient Safety and Risk Management Unit in Health Plans: Structure and Roles. Jerusalem: Ministry of Health; 2013 [Hebrew].

[17] Ministry of Health, Director General directive 09/2011, “Never Events”. Jerusalem: Ministry of Health; 2011 [Hebrew].

[18] Ministry of Health, Medicine Division directive 11/2012, The Duty of a Healthcare Institute to Report Special Events and Deaths. Jerusalem: Ministry of Health; 2012 [Hebrew].

[19] Cohen AD, Dreiher J, Regev-Rosenberg S, Yakovson O, Lieberman N, Goldfracht M, Balicer RD (2010). The quality indicators program in Clalit Health Services: the first decade. Harefuah.

[20] Ministry of Health (2019). National Program for Quality Indicators in the Community, Report for 2016–2018.

[21] Jaffe DH, Shmueli A, Ben-Yehuda A, Paltiel O, Calderon R, Cohen AD, Matz E, Rosenblum JK, Wilf-Miron R, Manor O (2012). Community healthcare in Israel: quality indicators 2007-2009. Isr J Health Policy Res.

[22] Calderon-Margalit R, Cohen-Dadi M, Opas D, Jaffe DH, Levine J, Ben-Yehuda A, Paltiel O, Manor O (2018). Trends in the performance of quality indicators for diabetes care in the community and in diabetes-related health status: an Israeli ecological study. Isr J Health Policy Res.

[23] Weisband YL, Krieger M, Calderon-Marglit R, Manor O (2020). Trends and socioeconomic disparities in diabetes prevalence and quality of care among Israeli children; 2011-2018. Isr J Health Policy Res.

[24] Podell R, Kaufman Shriqui V, Wolff Sagy Y, Manor O, Ben-Yehuda A (2018). The quality of primary care provided to the elderly in Israel. Isr J Health Policy Res.

[25] Ministry of Health (2006). National Program for Quality Indicators in the Community, Report for 2003-2005.

[26] Ministry of Health (2012). National Program for Quality Indicators in the Community, Report for 2008-2010.

[27] Ministry of Health. National Program for Quality Indicators in the Community, Report for 2011–2015, Available at https://48fc89f4-e14d-48de-bdc0-ec96de79873e.filesusr.com/ugd/76a237_8196a25144b843a78190e56a80cae1e1.pdf. Accessed 2 May 2020.

[28] Elhayany A (2001). Use of Medical Quality Indicators as a Performance Improvement Tool in Community Clinics.

[29] Bramesfeld A, Wensing M, Bartelsd P, Bobzina H, Greniere C, Heugrenf M, Jaffe Hirschfield D, Langeneggeri M, Lindelius B, Lucete B, Manore O, Schneideri T, Wardell F, Szecsenyi J. Mandatory national quality improvement systems using indicators: an initial assessment in Europe and Israel. Health Policy. 2016;120:1256–69. 10.1016/j.healthpol.2016.09.019 27793361

[30] Ministry of Health (2019). National Program for Quality Indicators in Hospitals, Report for 2013–2018.

[31] Ministry of Health (2020). The National Program for Quality Indicators in Hospitals, 2013–2020 indicators, version 01–2020-104.

[32] Peleg K, Rozenfeld M, Israeli A (2017). The danger of non-exhaustive quality measures: requiring hip fracture repair surgery within 48 hours – a case study. Isr J Health Policy Res.

[33] Davidson E. Leadership in Healthcare. Beer Sheva: Ben-Gurion University Publishing 2019. [Hebrew].

[34] Weinstein O, Cohen AD, Comaneshter D, Limoni Y, Hazanov I, Mishory-Deri M, Bitterman H, Codish S, Davidson E (2016). Community, hospital and in-between: Quality measures for the continuity of care. Harefuah.

[35] Rosen B, Ashkenzai Y, Ben-Assuli O, Karnieli-Miller O, Zelingher J, Hochman O, Rosenblum J. Organizational aspects of Israel’s national health information exchange: Smokler Center for Health Policy Research. Jerusalem: Myers-JDC-Brookdale; 2017. Available at https://brookdale.jdc.org.il/wp-content/uploads/2018/04/Eng_Summary_758-17.pdf.

[36] Abu-Saad K, Avni S, Kalter-Leibovici O. Health disparities monitoring in the U.S.: lessons for monitoring efforts in Israel and other countries. Isr J Health Policy Res. 2018;7:14. 10.1186/s13584-018-0208-1 PMC6389259 29490695

[37] Muhsen K, Green MS, Soskolne V, Neumark Y (2017). Inequalities in non-communicable diseases between the major population groups in Israel: achievements and challenges. Lancet..

[38] Ministry of Health (2019). Inequality in Healthcare and Dealing with it.

[39] Ministry of Health (2020). Inequality in Healthcare and Dealing with it.

[40] Balicer RD, Shadmi E, Lieberman N, Greenberg-Dotan S, Goldfracht M, Jana L, Cohen AD, Regev-Rosenberg S, Jacobson O (2011). Reducing health disparities: strategy planning and implementation in Israel’s largest health care organization. Health Serv Res.

[41] Balicer RD, Hoshen M, Cohen-Stavi C, Shohat-Spitzer S, Kay C, Bitterman H, Lieberman N, Jacobson O, Shadmi E (2015). Sustained reduction in health disparities achieved through targeted quality improvement: one-year follow-up on a three-year intervention. Health Serv Res.

[42] Spitzer-Shohat S, Shadmi E, Goldfracht M, Kay C, Hoshen M, Balicer RD (2016). Reducing inequity in primary care clinics treating low socioeconomic Jewish and Arab populations in Israel. J Public Health.

[43] Spitzer-Shohat S, Shadmi E, Goldfracht M, Kay C, Hoshen M, Balicer RD (2018). Evaluating an organization-wide disparity reduction program: understanding what works for whom and why. PLoS One.

[44] Brammli GS (2015). Inequalities in waiting times by socioeconomic status – a possible causal mechanism. Isr J Health Policy Res.

[45] Ministry of Health. Information System – Waiting times for Physicians in the Community. Available at https://gis.health.gov.il/waitingtime/. Accessed 2 May 2020.

[46] Bar-Ratson E, Dreiher J, Wirtheim E, Perlman L, Gruzman C, Rosenbaum Z, Davidson E (2011). The accreditation program in hospitals: Clalit Health Services experience. Harefuah.

[47] Joint Commission International (2020). Search for JCI accredited organizations.

[48] Kagan I, Farkash-Fink N, Fish M (2016). Effect of joint commission international accreditation on the nursing work environment in a tertiary medical center. J Nurs Care Qual.

[49] Ministry of Health (2019). Report on the Star Program to Improve Medical Care and Service in Neonatal Care Units.

[50] Ministry of Health (2020). Preventing Infections and Keeping Patients Safe.

[51] Nogueira-Jr C, Padoveze MC (2018). Public policies on healthcare associated infections: a case study of three countries. Health Policy.

[52] Ministry of Health. Infection Prevention and Control in Medical Institutes and Avoiding Antibiotics Resistance. Jerusalem: Ministry of Health Circular; 2012 [Hebrew].

[53] Ministry of Health, National Center for Infection Control (2019). The Incidence of Acquired CPE in Long-Term Care Facilities. Summary of a Decade of Intervention.

[54] Ministry of Health, National Center for Infection Control (2019). Acquired Sepsis in the Intensive Care Unit.

[55] Ministry of Health, National Center for Infection Control (2019). Annual Report 2018: Resistant Bacteria in General Hospitals.

[56] Ministry of Health, National Center for Infection Control (2019). Monitoring Acquired Urinary Tract Infections.

[57] Ministry of Health, National Center for Infection Control (2019). Administration of Antibiotics, General Hospitals in 2018.

[58] Krieger M, Abu Ahmad W, Wolff-Sagy Y, Horwitz E, Ben0=−Yehuda A, Paltiel O, Manor O, Calderon-Margalit R. Antibiotics use in Community healthcare in Israel as reflected in the national program for quality indicators. Harefuah 2019; 158:299–304. [Hebrew]. 31104389

[59] Ministry of Health (2016). Organizational Safety Culture Time Trends 2012–2015, General Hospitals Survey.

[60] Ministry of Health (2019). The National Model for Assessment of Patient Safety in General Hospitals, Report for 2017–2019.

[61] Ministry of Health (2016). Theory of Service and Patient Experience in the Israeli Healthcare System: Standard for Healthcare Organization – Professional Guide.

[62] Myers-JDC-Brookdale (2019). Findings from the 12^th^ survey of public opinion on the level of service and function of the Healthcare System.

[63] Gross R, Brammli Greenberg S, Mazliach R (2007). Public opinion on the level of services and function of the Israeli healthcare system, at the 10^th^ anniversary of the National Health Insurance law.

[64] Brammli Greenberg S, Medina-Hartum T, Belinsky A, Yaari A (2019). Public opinion on the level of services and function of the Israeli healthcare system in 2016.

[65] Ministry of Health (2019). Patient’s Experience Survey in the Emergency Department.

[66] Ministry of Health (2018). Patient's Experience Survey for Inpatient Services.

[67] Landon BE (2012). Physicians’ views of performance reports: grading the graders. Isr J Health Policy Res.

[68] Nissanholtz-Gannot R, Rosen B, The Quality Monitoring Study Group (2012). Monitoring quality in Israeli primary care: The primary care physicians’ perspective. Isr J Health Policy Res.

[69] Levi B, Zehavi A, Chinitz D (2018). Taking the measure of the profession: physician associations in the measurement age. Health Policy.

[70] Fisher M, Wagner O, Keinar T, Solt I (2015). Quality measures in medicine – a plea for new, value-based thinking. Harefuah.

[71] Paltiel O, Keidar Tirosh A, Paz Stostky O, Calderon-Margalit O, Cohen AD, Elran E, Valinsky L, Matz E, Krieger M, Ben Yehuda A, Jaffe DH, Manor O. Adherence to national guidelines for colorectal cancer screening in Israel: Comprehensive multi-year assessment based on electronic medical records. J Med Screen. 2020:969141320919152. 10.1177/0969141320919152. 10.1177/0969141320919152 32356670

[72] Tal O. Hospital accreditation – Time for rethinking? Harefuah. 2018;157:367–9 [Hebrew]. 29964376

